# Current Understanding of Pathogenic Mechanisms and Disease Models of Citrin Deficiency

**DOI:** 10.1002/jimd.70021

**Published:** 2025-03-27

**Authors:** Denis Lacabanne, Alice P. Sowton, Bosco Jose, Edmund R. S. Kunji, Sotiria Tavoulari

**Affiliations:** ^1^ Medical Research Council Mitochondrial Biology Unit University of Cambridge Cambridge UK

**Keywords:** citrin deficiency, disease models, mitochondrial transport, urea cycle disorders

## Abstract

Citrin deficiency (CD) is a complex mitochondrial disease with three different age‐related stages: neonatal intrahepatic cholestasis caused by CD (NICCD), failure to thrive and dyslipidemia caused by CD (FTTDCD), and type II citrullinemia (CTLN2), recently renamed adolescent and adult CD (AACD). While highly prevalent in the Asian population, CD is pan‐ethnic and remains severely underdiagnosed. The disease is caused by the dysfunction or absence of the mitochondrial aspartate/glutamate carrier 2 (AGC2/SLC25A13), also known as citrin. Citrin deficiency results in a direct impairment of the malate–aspartate shuttle and the urea cycle, with expected knock‐on effects on a multitude of other metabolic pathways, leading to a complicated pathophysiology. Here, we discuss our current knowledge of the molecular mechanism of substrate transport by citrin, including recent advances  suggesting against its calcium regulation. We also discuss the different types of pathogenic variants found in CD patients and new insights into their pathogenic mechanisms. Additionally, we provide a summary and assessment of the efforts to develop preclinical models as well as treatments for the disease.

## Graphical abstract

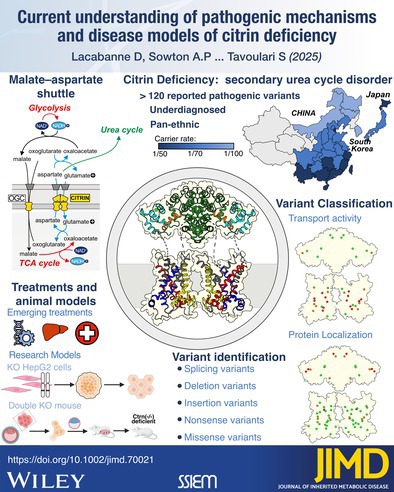

## 1 Introduction

**FIGURE 1:**
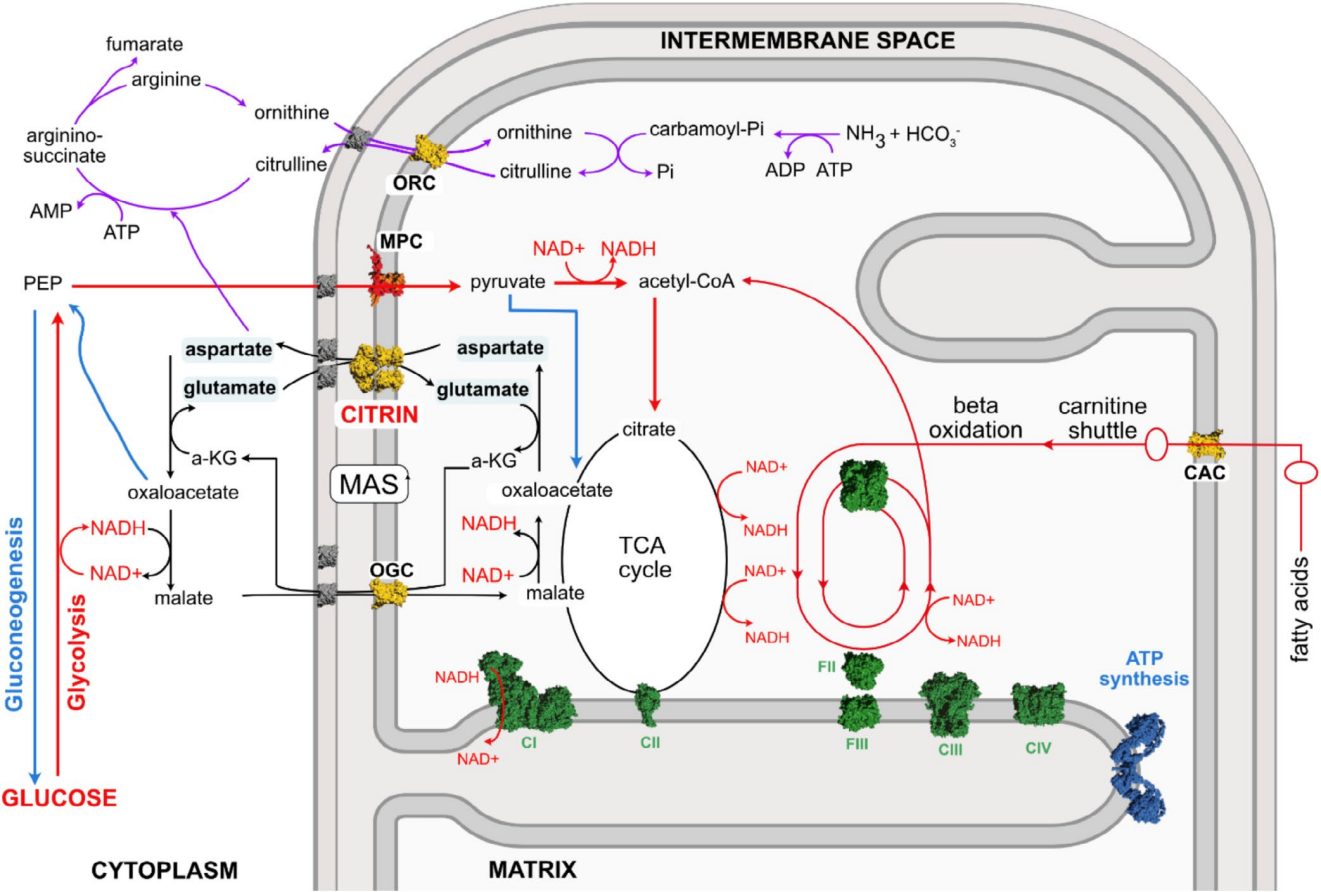
Metabolic pathways related to citrin function. Schematic representation of the malate–aspartate shuttle (black), glycolysis (red), tricarboxylic acid (TCA) cycle (black), gluconeogenesis (blue), ammonia fixation/urea cycle (purple) and fatty acid oxidation (red). Citrin is shown in yellow together with the oxoglutarate carrier (OGC), ornithine carrier (ORC) and the carnitine/acylcarnitine carrier (CAC). The mitochondrial pyruvate carrier heterodimer (MPC) is shown in red/orange. The respiratory chain complexes 1 to 4 (CI–CIV), acyl‐CoA dehydrogenases (FI), electron transfer flavoprotein (FII), and ETF‐ubiquinone oxidoreductase (FIII) are shown in green and the dimer of ATP synthase in blue. PEP: phosphoenolpyruvate, NAD: nicotinamide adenine dinucleotide. NAD^+^ and NADH are highlighted in red.

Citrin deficiency (CD) is an inborn error of metabolism and a secondary urea cycle disorder caused by pathogenic variants of the *SLC25A13* gene (OMIM #603859), encoding the mitochondrial aspartate/glutamate carrier 2 (AGC2), also called citrin [1, 2]. This carrier protein, located in the mitochondrial inner membrane, is responsible for the symport of glutamate with a proton into the mitochondrial matrix and the export of aspartate from the matrix to the cytoplasm [2, 3, 4, 5, 6, 7] (Figure 1). To date, more than 120 different pathogenic variants of citrin have been reported in the literature worldwide (reviewed in [8], with additional ones in Table 1) and 168 in the Human Gene Mutation Database (HGMD), but the number of identified variants continues to grow with an increasing number of diagnoses each year.

**TABLE 1:** Recently identified novel pathogenic variants.

Exon	Genotype	Protein prediction	Reference
Exon 3	c.173_174delTG	Val58fs81X	[9]
Intron 8	c.848+1G>T	Arg284fsX	[9]
Exon 11	c.1173T>G	Tyr391X	[9]
Exon 13	c.1307_1308delinsAA	Gly436Glu	[9]
Exon 16	c.1629dup	Ile544fs24X	[9]
Exon 7	c.674C>CA	Ser22X	[10]
Intron 8	c.848+6T>C	Arg284fsX	[11]
Exon 11	c.1043C>T	Pro348Leu	[12]
Exon 12	c.1216dupG	Ala406fs418X	[12]
Exon 3	c.135G>C	Leu45Phe	[12]
Exon 3/Intron2	c.70‐63_133del	Try24fs34X	[13]
Exon 18	c.[1956C>A;1962del]	[Gln652Lys;Phe654Lfs45X]	[13]
Exon 15	c.1591G>A	Gly531Ser	[14]
Exon 14	c.1402C>T	Val468Leu	[15]
Exon 15	c.1474C>T	Arg492Trp	[16]

Citrin is expressed variably across tissues, with the liver, kidney, heart, pancreas, and small intestine exhibiting the highest levels [17, 18, 19], whereas aralar (AGC1), a paralog of citrin encoded by *SLC25A12*, is primarily expressed in the brain, heart, and skeletal muscle [20]. Despite their common function, the different tissue distributions of citrin and aralar result in distinct clinical phenotypes associated with their pathogenic variants. While variants of *SLC25A13* cause CD, variants of *SLC25A12* lead to AGC1 deficiency, an infantile‐onset encephalopathy characterized by defective myelin synthesis and associated with hypotonia, arrested psychomotor development, and epilepsy [21, 22]. CD is considered to affect the liver primarily, whereas variants of *SLC25A12* do not result in hepatic phenotypes, likely due to the low aralar content in human livers, estimated in different studies to be either absent [23] or 13 times lower than citrin [24].

CD was first described six decades ago [25] and was originally characterized as both a rare and East Asia‐specific disease. It was first studied in Japan, where it is highly prevalent [26, 27], but is now recognized as being pan‐ethnic, with increasing numbers of patients of non‐Asian descent being diagnosed worldwide [9, 11, 28, 29, 30, 31, 32, 33, 34, 35, 36]. Additionally, CD is not as rare as previously thought, with an incidence of 1 in 17,000 births in Japan [37] and carrier rates measured at 1:41 in Singapore [38], 1:31 in Vietnam [39], and as high as 1:28 in the Southern regions of China [40].

Clinically, CD manifestations are diverse and show vast inter‐patient variability, as reviewed comprehensively elsewhere [34, 41, 42, 43]. Briefly, from birth, CD patients can present with neonatal intrahepatic cholestasis associated with CD (NICCD) (OMIM #605814), which is characterized by jaundice, cholestasis, hypoproteinemia, hypoglycemia, galactosemia, and multiple aminoacidemias, including citrullinemia [34]. Following NICCD, patients enter an adaptation stage, where they may experience a variety of milder clinical symptoms, typically accompanied by an aversion to carbohydrate‐rich food [44]. However, many patients at this stage suffer from failure to thrive and dyslipidemia caused by CD (FTTDCD), where they may suffer from growth impairment, fatigue, dyslipidemia, pancreatitis, and gastroenteropathy [35, 45, 46]. If undiagnosed or poorly managed, CD can escalate in a subset of patients, leading to the development of citrullinemia type II (CTLN2) (OMIM #603471) [27, 41], recently renamed as adolescent and adult CD (AACD; [34]). Often, this stage is characterized by frequent attacks of hyperammonemia, neuropsychiatric symptoms, and even brain edema, alongside a steatotic hepatic phenotype [34, 47]. However, it is unknown why AACD manifests only in a subset of patients and what triggers it. The precise molecular mechanisms driving these diverse phenotypes remain poorly understood, highlighting the need for more focused molecular research to unravel the links between genotype and clinical presentation.

Here, we review new insights into the molecular function of citrin, including how distinct pathogenic variants may be associated with discrete mechanisms of citrin dysfunction before considering current efforts in the development of preclinical models of CD in the hunt for effective treatments of the condition.

## 2 Citrin Sits at a Nexus of Metabolic Pathways

Citrin connects multiple metabolic pathways (Figure 1) and its pathogenic variants, as seen in CD, disrupt these pathways, leading to diverse phenotypes observed in patients. Citrin is an essential carrier protein of the malate–aspartate shuttle (MAS) [48], working alongside the mitochondrial oxoglutarate carrier (OGC, *SLC25A11*) (Figure 1). Whilst MAS involves a series of interconnected enzymatic reactions and transport steps, its net effect is redox shuttling, leading to NADH in the cytosol being oxidized and NAD^+^ in the mitochondrial matrix being reduced [49]. MAS therefore serves to regenerate cytosolic NAD^+^ for glycolysis, whilst translocating electrons produced during glycolysis into the mitochondrial matrix to feed into the electron transport chain and generate ATP. However, cytosolic NAD^+^ regeneration is vital beyond transferring energy equivalents between the cytosol and mitochondria, with many cytoplasmic reactions also being redox dependent, including serine biosynthesis, alcohol metabolism, and post‐translational modifications of proteins [50, 51, 52]. It is commonly assumed that many of the phenotypic manifestations of CD, such as fatigue, ultimately arise from elevation of the cytosolic NADH/NAD^+^ ratio as a result of MAS dysfunction [27]. However, this assumption has not been confirmed in patients, and the only evidence of altered NADH/NAD^+^ ratios in CD is derived from cultured cell models [23, 53] or inferred indirectly from lactate/pyruvate ratios in animal models [54, 55, 56, 57, 58].

In addition to maintaining redox balance, citrin contributes to the detoxification of ammonia, derived from the deamination of amino acids, via the urea cycle. The export of aspartate from the mitochondrial matrix into the cytosol by citrin is a key transport step required for the urea cycle in the liver (Figure 1). These reactions convert waste ammonia from protein breakdown into urea for excretion. Aspartate is required in the cycle in the intermediate step, combining with citrulline, itself exported from the mitochondria in exchange for ornithine by the mitochondrial ornithine carriers (ORC, *SLC25A2* and *SLC25A15*) to form argininosuccinate [59]. Citrin is critical for ensuring sufficient aspartate is available in the cytosol for continuous urea cycle flux. In CD, insufficient aspartate export is hypothesized to contribute to the accumulation of ammonia to toxic levels. Patients who progress to the most severe form of CD, AACD (previously CTLN2), display clinical symptoms associated with hyperammonemia, namely, neuropsychiatric symptoms, which can progress to coma and brain edema [43].

Also in the liver, aspartate is a key precursor for gluconeogenesis. As part of MAS, cytosolic aspartate aminotransferase converts aspartate to oxaloacetate. While oxaloacetate can be converted to malate, which is imported into the mitochondria through OGC as part of MAS, in the cytosol of hepatocytes, oxaloacetate can also be decarboxylated and phosphorylated by phosphoenolpyruvate carboxykinase. This reaction forms phosphoenolpyruvate for gluconeogenesis [60]. Mitochondrial oxaloacetate may also be exported as malate or phosphoenolpyruvate via other mitochondrial carriers. While other gluconeogenic substrates can be used for gluconeogenesis (alongside pyruvate itself), aspartate is considered to be one of the principal sources of cytosolic oxaloacetate for gluconeogenesis in the liver [61], highlighting the importance of citrin in this pathway. Therefore, CD might be associated with impaired hepatic gluconeogenesis. Alternatively, the hypothesized disturbances in cytosolic NADH/NAD^+^ ratio (caused by CD) have been proposed to impair gluconeogenesis by impeding the conversion of lactate to pyruvate and glycerol‐3‐phosphate to dihydroxyacetone phosphate. Impaired gluconeogenesis may contribute to hypoglycemia that some CD patients experience [62].

Citrin is essential for cellular homeostasis, supporting redox balance and energy metabolism via MAS, ammonia detoxification through the urea cycle, and glucose production by gluconeogenesis. Given these critical functions, disruption of citrin activity due to pathogenic variants can lead to the wide‐ranging metabolic disturbances observed in CD, underscoring the importance of the protein in both normal physiology and disease.

## 3 The Structure and Function of Citrin

### 3.1 Structural Model

**FIGURE 2:**
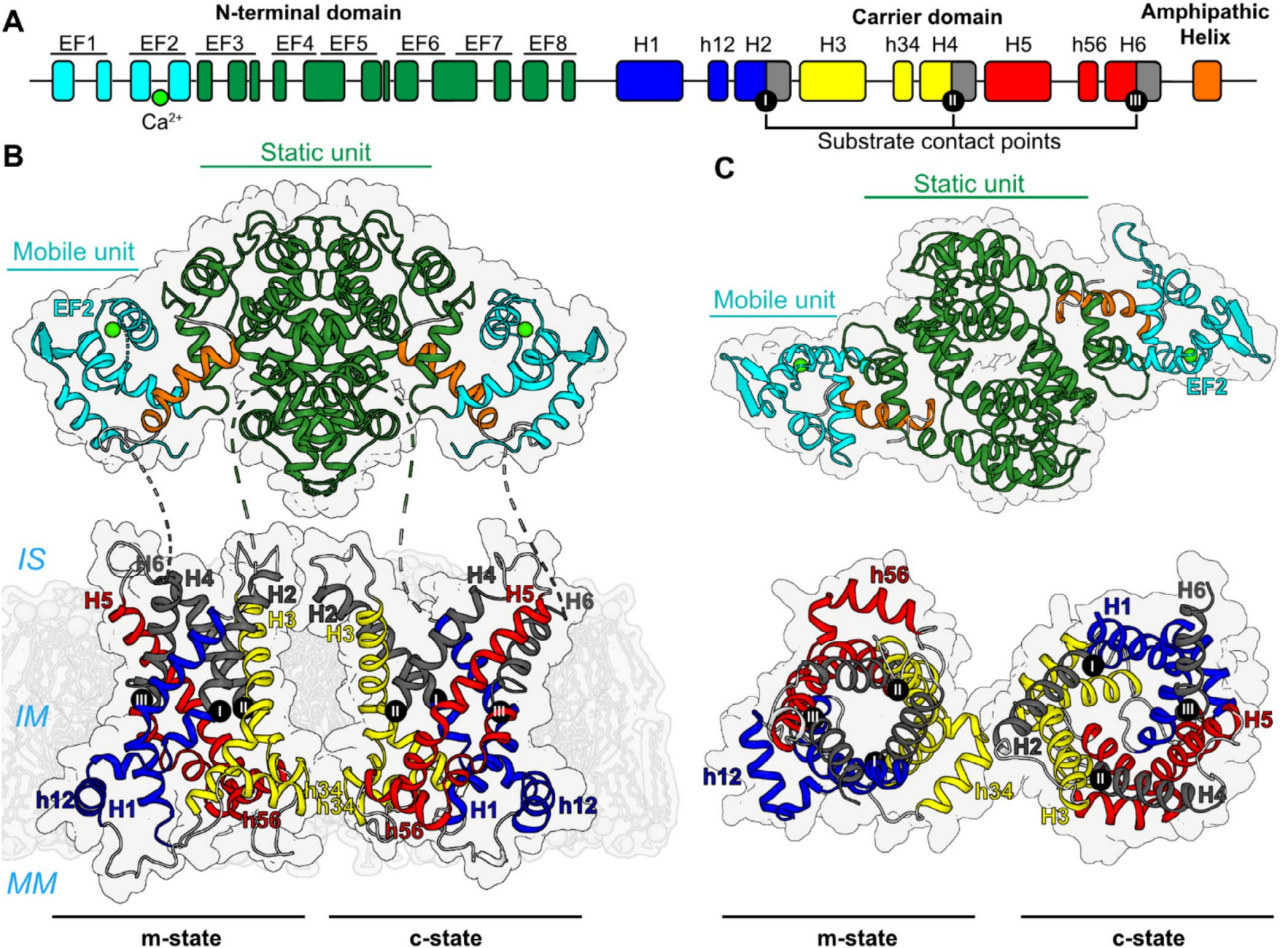
Structural model of citrin. (A) Domain structure of citrin. In the N‐terminal domain, the EF‐hand‐containing mobile unit and static unit are shown in cyan and green, respectively. The calcium bound in EF‐hand 2 is shown as a green sphere. In the carrier domain, the three core elements are shown in blue, yellow and red and the three gate elements in gray. The C‐terminal domain is shown in orange. (B, C) Lateral and cytoplasmic view of the citrin structural model in the same color scheme as in (A).

Citrin and aralar are unique members of the SLC25 family of mitochondrial carriers due to their structural complexity. They consist of three domains: an N‐terminal domain with eight EF‐hand motifs [2, 63], a mitochondrial carrier domain responsible for substrate transport [2], and a C‐terminal amphipathic α‐helix bound in a hydrophobic cleft within the N‐terminal domain [64] (Figure 2).

Unlike other SLC25 family members [65, 66, 67, 68], citrin and aralar form homodimers mediated by the N‐terminal domain [64]. EF‐hands 4–8 do not bind calcium but instead form a large dimerization interface of about 2000 Å^2^ [64]. It is likely that the two carrier domains in the dimer do not interact and function independently of each other [64, 65, 69].

The carrier domain of citrin and aralar resembles canonical SLC25 family members, such as the ADP/ATP carrier or uncoupling protein 1 [70, 71, 72]. It has a threefold pseudo‐symmetrical structure [73] formed by three sequence repeats [74]. Each domain consists of two transmembrane helices linked by a loop containing a matrix helix [73, 75], folding into a six‐helical bundle with a central water‐filled cavity [71, 72, 75] (Figure 2).

Whilst the atomic structure of full‐length citrin has not been resolved, a model has been proposed [8] (Figure 2) based on the structures of the N‐ and C‐terminal domains of citrin [64] and homology models of the carrier domain based on the ADP/ATP carrier [71, 72].

### 3.2 Transport Mechanism

Like other members of the SLC25 family, citrin operates via a ping‐pong transport mechanism [65], where glutamate and a proton have to be imported into the matrix before aspartate can be exported. This mechanism involves cycling between two conformational states, the “matrix‐open” state or “m‐state” in which the substrate binding site is open to the mitochondrial matrix, and the “cytoplasmic‐open” state or “c‐state” where it is open to the intermembrane space [71, 72, 76, 77] (Figure 2C). Glutamate and a proton bind to their binding sites in the c‐state [78, 79, 80], after which the carrier converts to the m‐state via an occluded state, which is closed to both sides of the membrane. After the substrates are released into the matrix, mitochondrial aspartate binds to the m‐state and triggers conformational changes, shifting the protein to the c‐state via an occluded state. Subsequently, aspartate can leave the binding site and diffuse into the intermembrane space, completing the exchange of substrates across the inner membrane of mitochondria. Although atomic details of this translocation mechanism of citrin are unavailable, the carrier domain of citrin shares all of the structural features previously proposed for the best‐studied example of this protein family, the ADP/ATP carrier [68, 71, 72, 81]. Recently, several missense pathogenic variants in residues involving the substrate binding site and key networks for state interconversions have been studied, showing detrimental effects on the transport activity and confirming the critical functional role of these residues (See Section 4; [69]). The roles of the N‐terminal domain and C‐terminal α‐helix remain unclear, though recent evidence shows that pathogenic variants at different positions of the N‐terminus interfere with protein biogenesis [69], also discussed in Section 4.

### 3.3 The Ambiguous Role of Calcium

**FIGURE 3:**
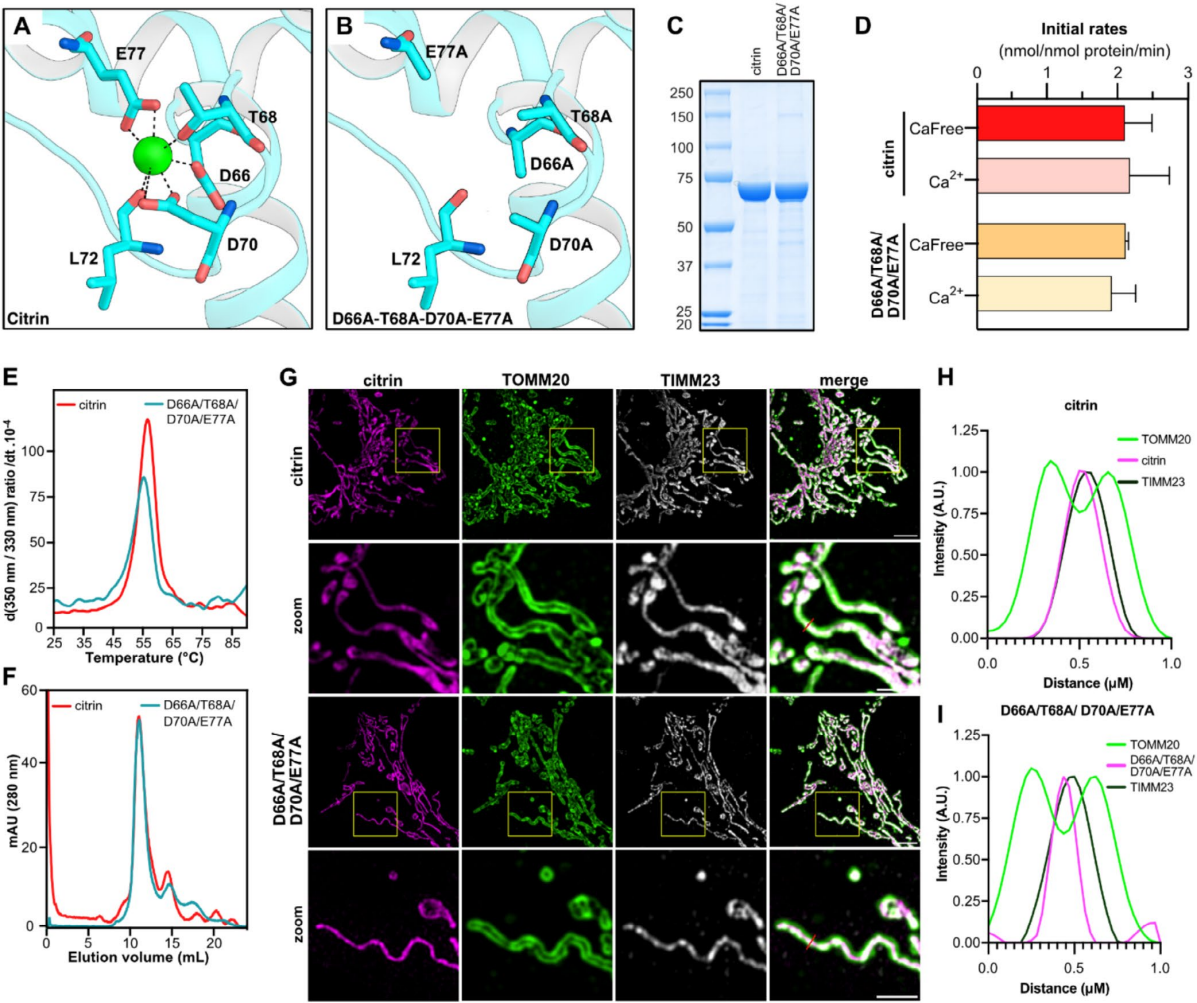
The transport activity of reconstituted citrin is not regulated by calcium. (A) The calcium‐binding site of citrin in the presence of calcium (green sphere). (B) The mutated calcium binding site in the D66A/T68A/D70A/E77A mutant. (C) Purification of wild‐type citrin and the D66A/T68A/D70A/E77A mutant for in vitro assays. (D) Initial rates of substrate transport for wild‐type citrin and D66A/T68A/D70A/E77A, in the presence or absence of calcium. (E) Thermostability analysis for wild‐type citrin and D66A/T68A/D70A/E77A. (F) Oligomeric state of wild‐type citrin and D66A/T68A/D70A/E77A via size exclusion chromatography. (G) Super‐resolution mitochondrial imaging of a HAP1 cell line, where both endogenous citrin and aralar are knocked‐out, expressing transiently wild‐type citrin or D66A/T68A/D70A/E77A. (H) Line‐scan analysis of the relative fluorescence intensity of TOMM20, citrin or D66A/T68A/D70A/E77A and TIMM23 staining for the selected area shown in (F). Figure adapted from Tavoulari et al. [69].

Several studies have previously shown that the malate–aspartate shuttle is activated by calcium, a property attributed to the mitochondrial aspartate/glutamate carriers [2, 63, 82, 83, 84, 85], which are known to bind calcium [86] at a single site, the preformed EF‐hand 2 3[64]; (Figure 3A). One caveat of these investigations is that the transport activity of the AGCs cannot be measured directly in a cellular or mitochondrial context. Instead, AGC activity is inferred from NADH oxidation measurements of MAS activity reconstituted from isolated mitochondria [63, 83, 84] or from metabolic changes that could be resulting from changes in MAS activity [2, 82, 85]. Some studies have also knocked down AGC to establish its direct involvement in the calcium effect on MAS [82, 83]. However, MAS is part of a complex metabolic network, involving the conversion of malate to oxaloacetate. This step is also a critical step in the tricarboxylic acid (TCA) cycle, which itself is upregulated by calcium [87, 88]. Thus, MAS and TCA cycle activities are critically linked, meaning an upregulation of the TCA cycle by calcium could lead to an increase in MAS activity, unrelated to changes in citrin activity.

In vitro studies with reconstituted purified citrin have also been employed to evaluate the above findings, but no consensus has been achieved so far. First, it was reported that reconstituted aralar1 from *Drosophila melanogaster* is activated by calcium in vitro [89]. In contrast, we have recently found that wild‐type human citrin activity, measured in proteoliposomes, was unaffected after calcium had been removed and replaced by magnesium ([69]; Figure 3C,D). Replacing calcium with another divalent cation had not been considered in previous in vitro activity measurements. However, this step is necessary, as cation‐free conditions can destabilize proteoliposomes, preventing the formation of concentration gradients required for transport activity, which can be mistaken for protein inactivation in the absence of calcium [69].

As achieving calcium‐free conditions is generally challenging and can create artifacts in proteoliposomes, another approach was employed to eliminate the calcium binding site by mutagenesis. We have shown that a mutant of human citrin (D66A/T68A/D70A/E77A), where the critical residues of the calcium binding site in EF‐hand 2 are mutated to alanine, rendering the protein unable to bind calcium, has transport activity comparable to wild‐type citrin ([69]; Figure 3A–D). These results contrast with previous studies where EF‐hand 1 and EF‐hand 2 mutagenesis in aralar resulted in decreased MAS activity, as measured by changes in NADH oxidation rates [83]. For human citrin, elimination of the calcium binding site did not affect the stability or oligomeric state of citrin (Figure 3E,F). Furthermore, the quadruple alanine mutant localized to the inner mitochondrial membrane, showing that calcium binding was not critical for biogenesis either (Figure 3G–I).

In structural studies, conformational changes of the N‐terminal domain of aralar alone have been observed in a calcium‐dependent manner [64], which could be consistent with a mechanism of calcium activation. However, these conformational changes were only observed in the absence of the amphipathic helix of the C‐terminal domain, which is not relevant in the context of the full‐length protein [64]. A structure of an N‐ and C‐terminal domain fusion of citrin, mimicking the correct structural context, could be obtained in a calcium‐bound state, but not in a calcium‐free state, despite the use of excess chelators [64], suggesting that the conformational changes might not occur in the presence of the amphipathic helix, independent of calcium. Interestingly, the structure of EF‐hand 2 in the N‐terminal domain structure of aralar is the same whether calcium is bound or not [64], suggesting that calcium binding or release cannot trigger conformational changes. Furthermore, EF‐hand structural mechanisms usually involve EF‐hand pairs, both capable of binding or releasing calcium; however, this is not the case in citrin. Thus, based on our functional and structural analyses of citrin, no functional role can be assigned to the bound calcium ion in EF‐hand 2. This suggests that calcium may not be playing a role in pathogenic mechanisms in citrin deficiency.

## 4 Pathogenic Variants of Citrin: More Than One Mechanism at Work

**FIGURE 4:**
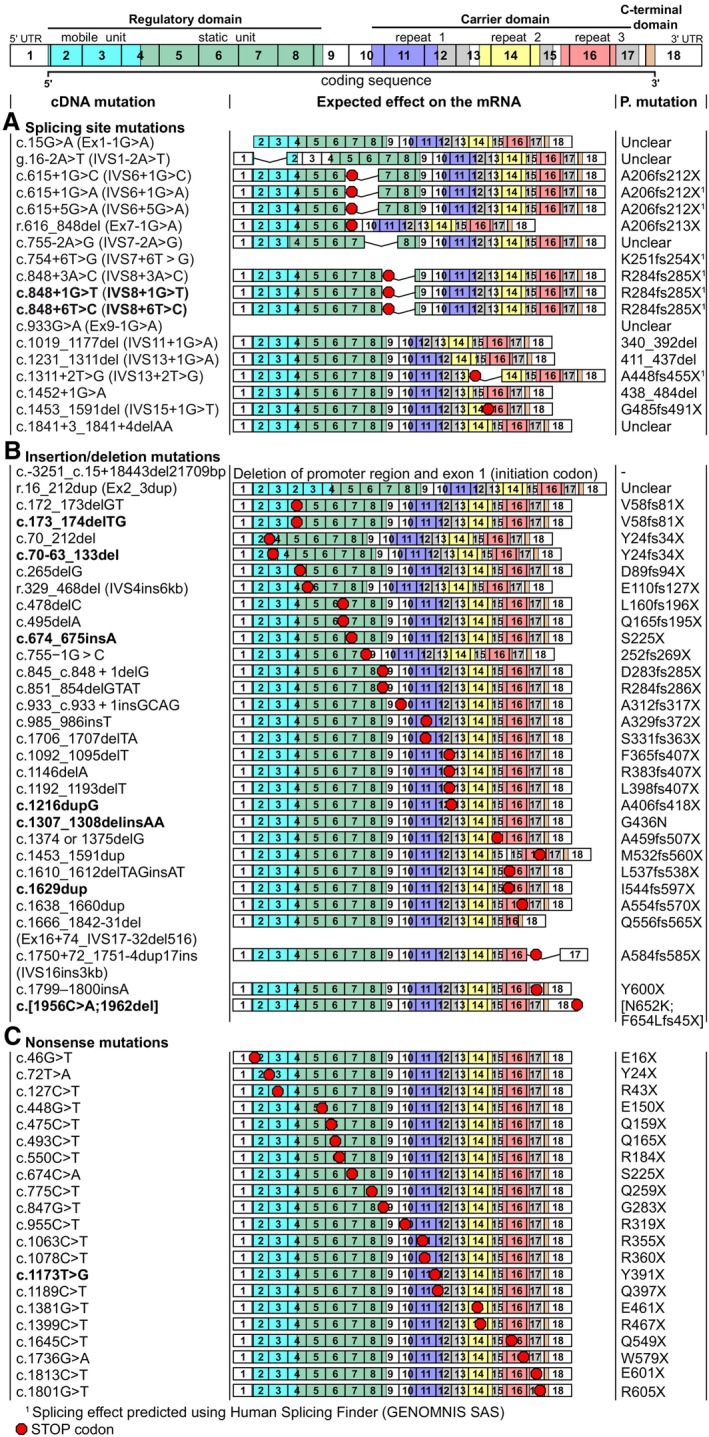
The wide range of pathogenic mutations of citrin. Positions of splicing, deletion, insertion, and nonsense variants reported in citrin deficiency, shown in the mRNA product (NM_014251.3). The red dots indicate the position of the substitutions. The color coding of the exons corresponds to the protein domains potentially translated, as shown in Figure 2. The potential translated protein outcome is indicated in the last column. The most recently reported variants (as in Table 1) are shown in bold. The effects of the splicing site variants were predicted using Human Splicing Finder [90].

**FIGURE 5:**
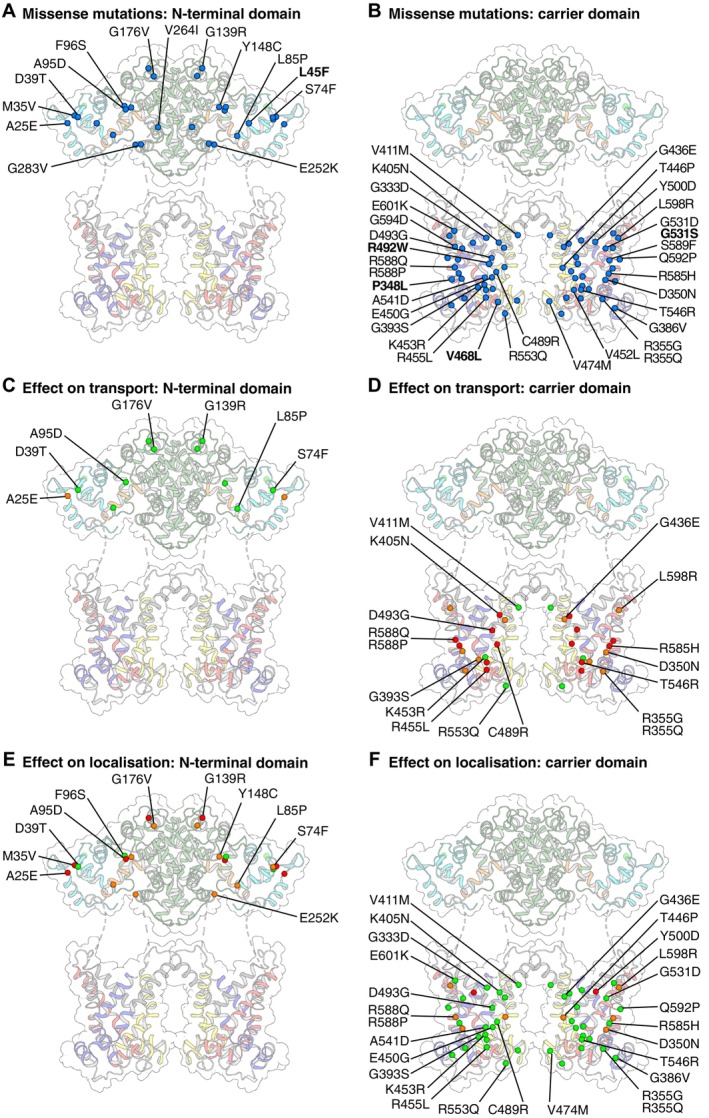
Effects of citrin missense variants on activity and mitochondrial localization. Positions of citrin missense variants in (A) the N‐terminal domain and (B) the carrier domain are highlighted in blue spheres. Novel variants most recently reported are shown in bold (also see Table 1). (C) Effect of citrin N‐terminal pathogenic variants on transport activity. (D) Effect of citrin carrier domain pathogenic variants on transport activity. For (C) and (D), residues with more than 50% activity are shown in green spheres, with 5%–50% activity with orange and with less than 5% activity in red. (E) Effect of citrin N‐terminal pathogenic variants on mitochondrial localization. (F) Effect of citrin carrier domain pathogenic variants on mitochondrial localization. In (E) and (F), mutants are hierarchically clustered for having primarily mitochondrial distribution (green spheres), mitochondrial distribution mixed with high levels of non‐mitochondrial (orange spheres) or primarily non‐mitochondrial distribution (red spheres).

Although CD is a monogenic disease, it involves a wide range of pathogenic variants of *SLC25A13*. Splice site variants, deletions, insertions, nonsense, and missense variants have all been reported (Figures 4 and 5; Table 1) (also reviewed in [8]), with more pathogenic variants in citrin reported than in all other mitochondrial carrier proteins combined [81]. Compound heterozygous patients are more common compared with patients carrying two copies of the same pathogenic allele, representing 53% of CD patients [8].

The most prevalent pathogenic allele is c.851_854delGTAT, a deletion variant found in more than 50% of patients in China and in approximately 30% of patients in Japan and Korea [91, 92]. Other highly prevalent pathogenic alleles contain splice site variants [40, 91, 92], with prevalence ranging from 48% of all pathogenic alleles in Japan to 24% in China. Approximately one‐third of the known splice site variants in the *SLC25A13* gene occur in introns 6 and 7 (1.8 and 1.4 kb, respectively). These variants lead to aberrant splicing, introducing premature stop codons and resulting in truncated proteins. Nonsense variants are less prevalent, representing only around 4% of Chinese patients [8].

Most splice site, deletion, insertion, or nonsense variants disrupt the citrin carrier domain, which is incompatible with these variants being functional transporters. However, in many variants, the mRNA may undergo nonsense‐mediated decay [93], potentially abolishing protein expression altogether, although direct experimental or clinical data have not been available to support this hypothesis in the majority of cases [94].

Missense variants represent 20% of Chinese CD cases. To date, more than 50 missense variants have been reported, of which about 70% are located in the carrier domain of citrin, with the remaining located in the N‐terminal domain (Figure 5A,B). It is expected that these variants produce full‐length protein products, and while limited data are available, normal expression levels have been reported for G437E in fibroblasts of a heterozygous patient [31] and for A25E in a homozygous patient [30].

Recently, we have proposed that there are distinct mechanisms of dysfunction elicited by different missense variants in citrin (Figure 5C–F) [69], which may be relevant to the broad phenotypic landscape observed in CD patients. In in silico and in vitro tests of 33 missense variants, most of those investigated in the carrier domain led to severely impaired substrate transport activity. Complete or near‐complete loss of transport activity was measured when variants occurred in functional elements of the carrier, known to be crucial for substrate translocation in other members of the SLC25 family, such as the putative substrate binding site (K405N, D493G and R588Q) and the cytoplasmic or matrix networks (D350N, C489R, T456R). As an exception, conservative substitutions, like K453R in the matrix network, reduced activity but did not inactivate the protein completely. Severe effects on transport activity were reported for several additional substitutions in the carrier domain at structurally important positions or residues important for dynamic changes. While activity was severely affected for most variants in the carrier domain, the expression and mitochondrial localization of most of these variants were unaffected [69].

Conversely, most N‐terminal missense variants tested retained high levels of transport activity, and some, as for example L95D, were comparable to wild‐type citrin. However, the majority of the tested N‐terminal domain variants interfered with the ability of the protein to localize to the mitochondria, despite being positioned in different regions of the N‐terminal domain [69]. Therefore, it appears that pathogenic variants in the N‐terminal domain interfere with mitochondrial localization, suggesting that it plays a critical role in the biogenesis of citrin, although further evidence is necessary to support this hypothesis.

## 5 Therapies and Models of Citrin Deficiency

While the dysfunction of citrin manifests in a complex clinical phenotype across different life stages, therapeutic options remain limited, and no current treatment addresses the underlying metabolic dysregulation comprehensively. To bridge this gap, effective preclinical models have become essential for evaluating therapeutic strategies aimed at restoring metabolic homeostasis. Here, we discuss published efforts to develop preclinical models of the disease, as well as therapies used or under investigation. Future directions for developing treatments and further understanding of the pathology have been reviewed elsewhere [95].

### 5.1 Cell Models

**FIGURE 6:**
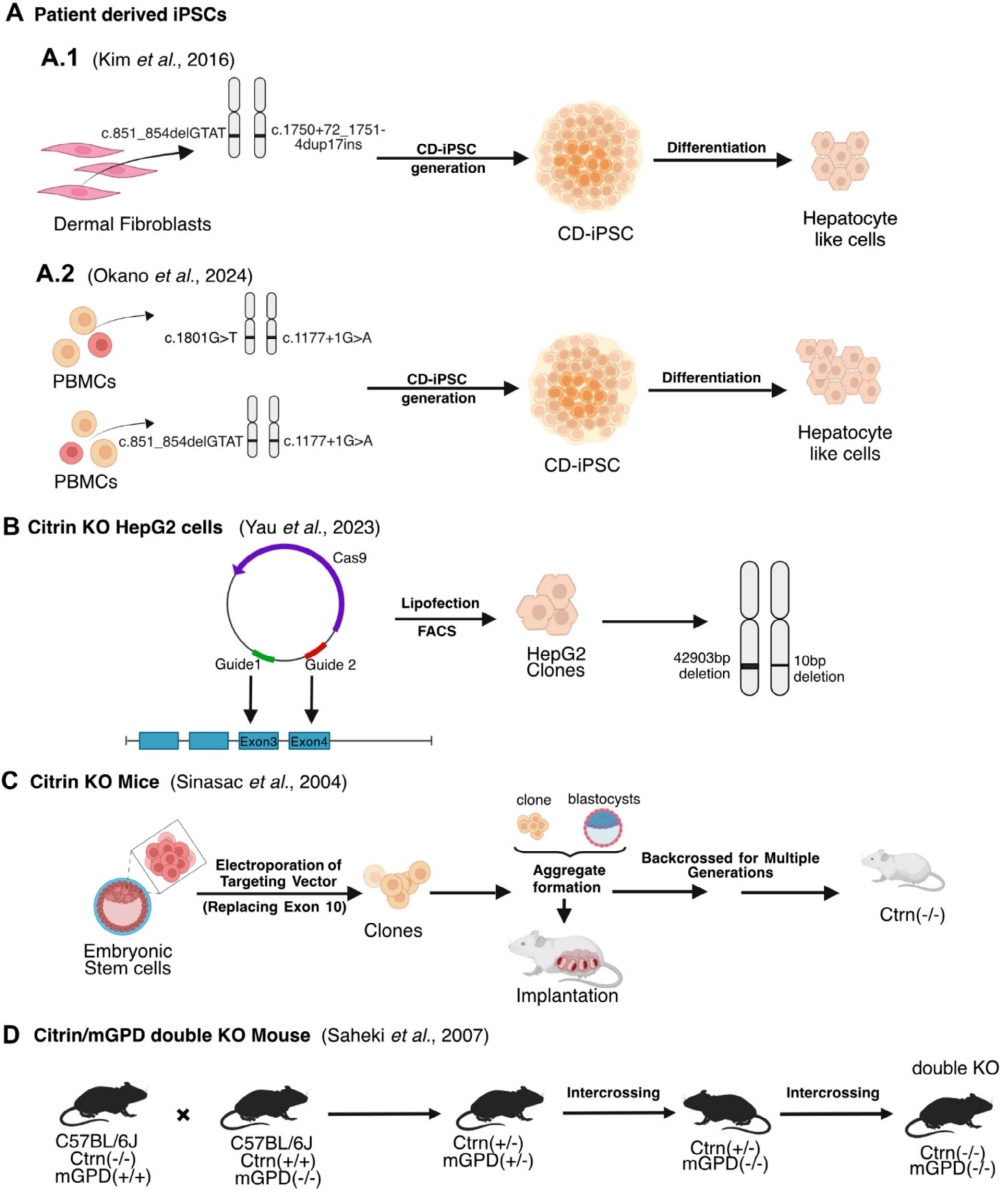
Generation procedures of cellular and mouse models of citrin deficiency. With the exception of patient‐derived induced Pluripotent Stem Cells (iPSCs), model design efforts have focused on knocking out the citrin gene. Ctrn, citrin; FACS, fluorescence‐activated cell sorting; KO, knock out; mGPD, mitochondrial glycerol‐3‐phosphate dehydrogenase; PBMCs, peripheral blood mononuclear cells. Image created with BioRender.com.

The first cellular models of CD were developed with human induced pluripotent stem cells (hiPSCs) derived from dermal fibroblasts of a CD patient carrying the c.851_854delGTAT and c.1750+72_1751‐4dup17ins (IVS16ins3kb) variants [96] (Figure 6). Subsequently, these hiPSCs were differentiated into hepatocyte‐like cells and demonstrated lower rates of ureagenesis from NH_4_Cl compared with hepatocyte‐like cells generated from hiPSCs of non‐CD patients, indicating that they recapitulate the urea cycle defects observed in CD. This finding has recently been recapitulated in hepatocyte‐like cells derived from hiPSCs developed from peripheral blood mononuclear cells of two CD patients (patient 1: c.1177+1G>A and c.1801G>T, patient 2: c.1177+1G>A and c.851_854delGTAT) [97]. The CD‐hiPSCs developed by Kim et al. also showed increased triacylglycerol accumulation under high‐glucose conditions, linked to reduced expression of PPARα and its target genes previously indicated in CD [98], as well as downregulation of mitochondrial marker genes, such as TOM20 and Parkin [96]. This patient‐derived model system, therefore, provides evidence for aberrant regulation of fatty acid metabolism via PPARα and mitochondrial abnormalities as a central mechanism in the fatty liver phenotype of CD. However, the cells in the study of Kim et al. are derived from only one patient, carrying two indel variants. Whether these pathophysiological mechanisms are applicable to CD as a whole, given the numerous distinct variants associated with it, remains to be investigated.

More recent work has used HepG2, a human hepatoblastoma cell line [99], and CRISPR/Cas9 gene editing to create an *SLC25A13* knockout via targeted deletion of a ~43 kb region spanning exons 3 and 4 in one allele, with a serendipitous 10 bp deletion in the second allele [53] (Figure 6). Yau et al. proposed that glycolytic capacity, fatty acid oxidation, and mitochondrial respiratory activity are all impaired in the citrin‐deficient cells, whilst proteins and genes involved in lipogenesis, cholesterol, and bile acid metabolism are upregulated. Supplementing the cells with nicotinamide riboside, an NAD^+^ precursor [100], reversed several phenotypic abnormalities, suggesting its potential as a treatment for CD.

However, this system requires further validation as some fundamental issues have not been addressed. First, HepG2 cells carry chromosomal abnormalities [101, 102, 103], including, importantly for CD research, trisomy of chromosome 2, on which aralar and mitochondrial glycerol‐3‐phosphate dehydrogenase (mGPD) are located. Whether HepG2 cells show elevated levels of aralar expression and function, as has been suggested previously [104], needs to be evaluated carefully as it will influence whether citrin models need to be initiated on a wild‐type or aralar‐suppressed background. Aralar upregulation in HepG2 cells has also been suggested to result from epigenetic upregulation in culture [104] and thus the impact of epigenetic regulation also needs further investigation. mGPD is a key enzyme for the glycerol phosphate shuttle, which provides an alternative route for transferring energy equivalents between the mitochondria and cytosol. Trisomy of chromosome 2 may therefore not only contribute to elevated aralar but also elevation of mGPD, and possibly reduce reductive stress in CD models.

Second, some other known genomic abnormalities may have functional implications for evaluating treatments for CD in an in vitro CD model. HepG2 cells have been shown to lack two key enzymes of the urea cycle (namely arginase 1 and ornithine transcarbamylase), diminishing the capacity of wild‐type HepG2 to produce urea from NH_4_Cl [105]. Therefore, the evaluation of treatments to ameliorate the secondary urea cycle disorder requires the re‐establishment of a functional urea cycle in HepG2. Beyond differences in the urea cycle, HepG2 cells exhibit known metabolic differences compared with primary hepatocytes, including altered insulin‐sensitive glucose metabolism [106].

Finally, the work by Kim et al. highlighted that there may be differences in mitochondrial content between CD patient cells and healthy controls, alongside differences in mitochondrial morphology [96]. Therefore, any measurement of mitochondrial respiratory activity or metabolite level needs to be carefully normalized to the number of cells actually respiring in any particular assay. Growth rates between citrin knockout cells and controls are likely to be different. Ideally, normalization should also include a marker of mitochondrial content [107] to account for any differences in mitochondrial density as a result of the CD phenotype. Thus, it is still unknown whether there is an underlying deficit in mitochondrial respiratory function in these HepG2 citrin‐deficient cells, or whether the observed phenotype is merely a result of reduced cell number and mitochondrial density related to the citrin deficiency.

### 5.2 Animal Models

Efforts to develop an animal model of CD were initiated decades ago, focusing on mice (Figure 6). As with many monogenic diseases, a knockout mouse was first generated. However, despite showing a reduction in hepatic MAS activity through the loss of the primary hepatic isoform of AGC, the citrin‐KO mouse showed no phenotypic similarities to either AACD (previously CTLN2) or NICCD, even when mice were aged up to 12 months [58]. The lack of a CD phenotype was attributed to the high level of activity of the glycerol phosphate shuttle in murine livers compared with humans, circumventing the disruption of the MAS [108, 109]. Thus, a double KO mouse was generated, with genes for both citrin and mGPD disrupted, to circumvent the limitations of the citrin‐KO model [56]. This citrin‐mGPD double KO mouse presented growth retardation, hyperammonemia, hepatic triacylglycerol accumulation, as well as a similar aversion to carbohydrate‐rich foods and alcohol seen in CD patients and reduced food intake when fed a carbohydrate‐rich, protein‐poor rodent chow [110, 111]. However, there are several caveats to using this double KO mouse as an animal model of CD. Firstly, mGPD remains active in human pathology, and, whilst the activity of the glycerol‐phosphate shuttle is reportedly low in the human liver [108], it remains a mechanism of transport for reducing equivalents in and out of the mitochondria. Further, whether the shuttle activity compensates for the disruption of the MAS in CD is unknown. Additionally, single mGPD KO mice show phenotypic alterations that may confound the results of interpreting those of a double KO mouse, including impaired weight gain, lower plasma glucose, and altered urea cycle enzyme activity [56, 112, 113]. An additional problem with the double KO mouse was that it was generated on an inbred mouse strain background (C57Bl/6J), unlike the citrin‐KO mouse, which was generated on a mixed genetic background. Saheki et al. generated the double KO mouse in the C57Bl/6J background due to the presence of a mild phenotype with the citrin‐KO mouse (characterized by mild hypoglycemia and fatty liver) when it was congenic on the C57Bl/6J background [56]. The C57Bl/6J mice, however, carry known genetic polymorphisms and mutations affecting metabolism and glycemic tolerance compared with other mouse strains [114]. This includes a 17,814 bp deletion in the *Nnt* gene, encoding nicotinamide nucleotide transhydrogenase. This gene is crucial for mitochondrial NADPH regeneration, and its loss disrupts glucose homeostasis [114]. These disruptions are likely to exacerbate mitochondrial redox potential issues caused by citrin‐mGPD knockouts [115].

### 5.3 Treatments and Biomarkers Proposed Through CD Models

Despite the caveats, both mouse models have provided insights into CD and potential treatments and biomarkers. Elevated hepatic glycerol‐3‐phosphate has been identified as a key metabolic signature in the double KO mouse, which shows further hepatic accumulation following an oral sucrose challenge [111, 116]. Oral sucrose administration has also been shown to result in increased hepatic citrulline content, with decreased citrate and malate concentrations, both attributed to inhibition of ureagenesis and the TCA cycle, respectively [116]. Glycerol‐3‐phosphate has also been proposed to be a potential diagnostic marker of CD, alongside glycerol, based on the accumulation of these metabolites in the urine of the double KO mouse [54]. Urine glycerol and glycerol‐3‐phosphate were also found to accumulate in CD patients aged 1–9 years [54], highlighting that these metabolites may indeed be useful diagnostic markers for CD, especially in the silent period where few other clinical symptoms are present [117].

Both metabolic and genetic therapies have been proposed for the treatment of CD and have been investigated using both the citrin‐deficient [23, 55] and double KO mouse [57, 110, 118]. Using in situ liver perfusion, Moriyama et al. tested the individual effects of pyruvate, aspartate, and citrate to improve ureagenesis from ammonia in citrin‐deficient mice. This analysis showed that a hepatic perfusion of pyruvate at millimolar concentrations led to an increase in the rates of ureagenesis from NH_4_Cl in the citrin‐deficient liver to levels seen in the wild‐type, whilst aspartate and citrate were unable to bring about this effect [55]. However, the inability to achieve plasma concentrations of pyruvate to those shown to be necessary by Moriyama et al. makes pyruvate therapy an unlikely target for CD treatment. Through the addition of various supplements to a purified rodent diet lower in protein and higher in carbohydrate than standard laboratory chow, Saheki et al. demonstrated that increased dietary protein content improved food intake and weight gain in the double KO mouse [110]. Supplementing the diet with alanine, sodium glutamate, or MCTs improved food intake and weight gain in the double KO mouse, whilst also suppressing the hepatic increase in glycerol‐3‐phosphate and citrulline following an enteral sucrose challenge, although to a lesser extent with MCT supplementation [110]. Similarly, oral supplementation with ornithine, combined with either alanine or aspartate, has been shown to suppress the elevation in blood ammonia and plasma citrulline in the double KO mouse, following a combined sucrose‐glycine challenge [57]. Taken together, these studies highlight that the natural avoidance of carbohydrate‐rich foodstuffs in preference for diets richer in protein and fat content by patients may have a molecular basis in minimizing the risk of hyperammonemia and citrullinemia associated with CD. Patients may therefore be “self‐supplementing” their diets in a manner to minimize potentially pathological phenotypes associated with CD [44]. Optimizing dietary protocols for the stages of CD remains an active area of research.

Additional treatments, including gene therapies, are being explored in these models (also reviewed in [119]). González‐Moreno et al. used the *Ctrn* ^−/−^ mouse to show that aralar can functionally replace citrin and therefore could serve as a potential gene therapy in CD [23]. Primary hepatocytes from *Ctrn* ^−/−^ mice showed normalized cytosolic NADH/NAD^+^ ratios when transfected with exogenous aralar, whilst hepatic mitochondria expressing exogenous aralar under a liver‐specific promoter showed a small increase in the activity of the MAS, as measured by NADH decay rate in isolated mitochondria [23]. This approach provides a potential therapeutic route by which aralar could replace the mutated citrin protein in CD. However, whether aralar can ameliorate any of the pathological symptoms of CD remains undetermined, as does the interplay between exogenous aralar overexpression when residual citrin activity is present, as is the case with some missense variants in CD.

As an alternative approach to treating citrin deficiency, codon‐optimized human citrin (hCitrin) mRNA encased in lipid nanoparticles (LNPs) has been shown to achieve a small but significant increase in hepatic citrin expression (2%–5% of wild‐type) in the *Ctrn* ^−/−^ mouse 24 h following intravenous administration [118]. Furthermore, double KO mice receiving three weekly intravenous injections of hCitrin‐mRNA‐LNPs showed an increased preference for voluntary sucrose consumption, indicative of a reduction in the canonical aversion to carbohydrate‐rich foods associated with CD [118]. Following an oral sucrose challenge, hCitrin‐mRNA‐treated double KO mice further showed hepatic citrulline and plasma ammonium concentrations lower than those of double KO mice treated with a control mRNA, with concentrations of these metabolites maintained at levels similar to those measured in wild‐type mice [118]. Although it is still unclear how such a low expression of citrin can ameliorate the clinical phenotype, these results suggest that mRNA therapy, delivered via LNPs, may be a promising avenue to explore as a treatment strategy for CD. Several questions remain, such as which levels of citrin expression would be required to reverse the disease phenotype at different stages. Another question is whether introducing a full‐length protein transcript into patients with severe truncations would result in immunological reactions.

Despite the clear outstanding questions, clinical guidelines for the management and treatment of CD are comprehensively evaluated in several recent reviews [62, 120, 121].

## 6 Conclusion

CD represents a complex metabolic disorder, encompassing a wide range of genetic variants that impact key metabolic processes, particularly in the liver. The variable clinical presentations of the disease, spanning from neonatal intrahepatic cholestasis to adult‐onset citrullinemia type II, underscore the intricacy of the role of citrin in cellular metabolism and the challenges inherent in devising targeted therapeutic strategies. Whilst progress has been made in elucidating the structural and functional implications of various pathogenic variants, the precise mechanisms by which these variants disrupt metabolic homeostasis remain incompletely understood. Refined preclinical models that more accurately mimic the human pathology at different disease stages are required to elucidate the molecular mechanisms underlying CD. Dietary modifications, gene therapy, and mRNA‐based treatments offer promising avenues, but in order to take advantage of these technologies, reliable disease models will also be key. Addressing these gaps will be crucial for transforming our understanding of CD into effective, patient‐centered treatments that can improve quality of life and long‐term outcomes significantly for those affected by this challenging disorder.

## Author Contributions

**D.L., A.P.S.** and **S.T.:** writing original draft. **D.L., A.P.S., B.J., E.R.S.K.,** and **S.T.:** writing, reviewing, and editing. **D.L.** and **B.J.:** visualization. **E.R.S.K., S.T.:** supervision.

## Ethics Statement

The authors have nothing to report.

## Conflicts of Interest

The authors declare no conflicts of interest.
